# OASIS 2: online application for survival analysis 2 with features for the analysis of maximal lifespan and healthspan in aging research

**DOI:** 10.18632/oncotarget.11269

**Published:** 2016-08-12

**Authors:** Seong Kyu Han, Dongyeop Lee, Heetak Lee, Donghyo Kim, Heehwa G. Son, Jae-Seong Yang, Seung-Jae V. Lee, Sanguk Kim

**Affiliations:** ^1^ Department of Life Sciences, Pohang University of Science and Technology, Pohang, Korea; ^2^ Department of IT Convergence and Engineering, Pohang University of Science and Technology, Pohang, Korea; ^3^ School of Interdisciplinary Bioscience and Bioengineering, Pohang University of Science and Technology, Pohang, Korea; ^4^ EMBL/CRG Systems Biology Research Unit, Centre for Genomic Regulation (CRG), Barcelona, Spain

**Keywords:** web-service, statistics, survival, maximum lifespan, healthspan

## Abstract

Online application for survival analysis (OASIS) has served as a popular and convenient platform for the statistical analysis of various survival data, particularly in the field of aging research. With the recent advances in the fields of aging research that deal with complex survival data, we noticed a need for updates to the current version of OASIS. Here, we report OASIS 2 (http://sbi.postech.ac.kr/oasis2), which provides extended statistical tools for survival data and an enhanced user interface. In particular, OASIS 2 enables the statistical comparison of maximal lifespans, which is potentially useful for determining key factors that limit the lifespan of a population. Furthermore, OASIS 2 provides statistical and graphical tools that compare values in different conditions and times. That feature is useful for comparing age-associated changes in physiological activities, which can be used as indicators of “healthspan.” We believe that OASIS 2 will serve as a standard platform for survival analysis with advanced and user-friendly statistical tools for experimental biologists in the field of aging research.

## INTRODUCTION

The amount of lifespan data is increasing due to rapid growth in the field of aging research. Hence, proper statistical analysis for dissecting the genetic and environmental factors associated with aging has become more crucial than ever before. We previously reported OASIS, a web service for survival analysis [1]. OASIS has become a popular online platform for experimental biologists in the field of aging research who need convenient access to statistical tools for the analysis of survival data including lifespan. After 5 years of extensive OASIS usage, it has become apparent that more features are required for the analysis of complex phenomena involved in aging. Here, we report OASIS 2, which provides crucial updated features including advanced statistical analysis and an enhanced user-friendly interface.

## RESULTS

OASIS 2 has a new feature for the proper quantification of differences in maximal lifespan between datasets. The maximal lifespan is the upper percentile of the lifespan distribution, which contrasts with the mean lifespan [2]. The maximal lifespan is determined by the “fundamental process of aging,” whereas the mean lifespan changes with various conditions such as diseases. This is of interest, because increasing the maximal lifespan can imply that an intervention is slowing the general processes of aging and not merely retarding the development of specific diseases [3]. Hence, it could be useful to detect differences in maximal lifespan as opposed to simply the “curve squaring” that can be induced by increasing the mean or median lifespan without increasing the maximal lifespan [4].

**Figure 1:**
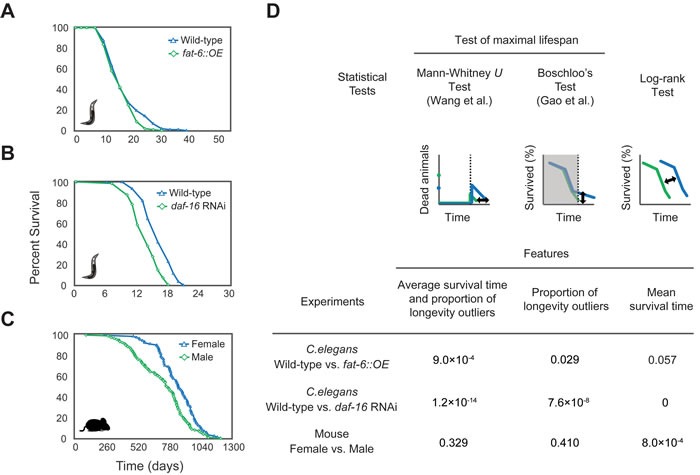
Analysis of maximal lifespans using OASIS 2 Survival plots of A wild-type and *fat-6*-overexpressing *C*. *elegans*, **B.** wild-type and *daf-16* RNAi-treated *C. elegans* [7], and **C.** female and male wild-type mice visualized using OASIS 2 [8]. **D.** Shown are the conceptual figures of three statistical tests. The *P*-values of each statistical test are shown.

Using the maximal lifespan analysis implemented in OASIS 2, we tested *Caenorhabditis elegans* lifespan data that have different maximal lifespan values (Materials and Methods). Worms overexpressing *fat-6*, which encodes delta(9)-fatty-acid desaturase, had a mean lifespan similar to that of wild-type worms but had a shorter maximal lifespan (Figure 1A). The log-rank test that was implemented in our previous version of OASIS showed that the difference between the wild-type and transgenic animals was not statistically significant (*P* = 0.057; Figure 1D). In contrast, Boschloo's test, which is implemented in OASIS 2 as well as in SurvCurv [5] and examines the differences in the proportion of longevity outliers that live beyond specific time points, detected a significant difference in maximal lifespan between the wild-type and transgenic animals (*P* = 0.029; Figure 1D). In addition, a Mann-Whitney *U* test, as modified by Gao and Allison [6], which was newly implemented in OASIS 2, showed that the difference in maximal lifespan was highly significant (*P* = 9.0×10^−4^; Figure 1D). The modified Mann-Whitney *U* test was able to determine the differences in the distribution tails of survival data affecting the maximal lifespan as well as the differences in the proportions of longevity outliers. To the best of our knowledge, OASIS 2 is the only web service that provides such functionality.

We also analyzed other lifespan datasets taken from different conditions and model organisms. For example, worms treated with an RNAi targeting *daf-16*, FOXO transcription factor, had shorter mean lifespan and maximal lifespan than wild-type worms (Figure 1B) [7]. A log-rank test, Boschloo's test, and the modified Mann-Whitney *U* test indicate that the differences in the mean and maximal lifespans are significant (*P* = 0 in the Log-rank test, *P* = 7.6×10^−8^ in Boschloo's test, *P* = 1.2×10^−14^ in the Mann-Whitney *U* test; Figure 1D). We re-analyzed mouse lifespan data that had similar maximal lifespan values [8]. Female mice had a maximal lifespan similar to that of male mice but had a longer mean lifespan (Figure 1C). A log-rank test indicates that the difference in mean lifespan values is significant (*P* = 8.0×10^−4^; Figure 1D); however, the Boschloo's test and the modified Mann-Whitney *U* test show that the difference in maximal lifespan values is not significant (*P* = 0.329 in the Mann-Whitney *U* test; *P* = 0.41 in Boschloo's test; Figure 1D). We also validated the statistics by comparing the results to those of (a) SurvCurv, which provides comparisons of maximum lifespans, and (b) JMP and STATA, which are well-known statistical packages. We confirmed that the OASIS 2 results are the same as the results obtained by SurvCurv, JMP, or STATA. We provide the comparison of those statistical data as well as the raw data on the OASIS 2 website (http://sbi.postech.ac.kr/oasis2/benchmark). Overall, OASIS 2 provides analytical methods that are suitable for calculating the statistical significance of survival data focused on maximal lifespan.

**Figure 2:**
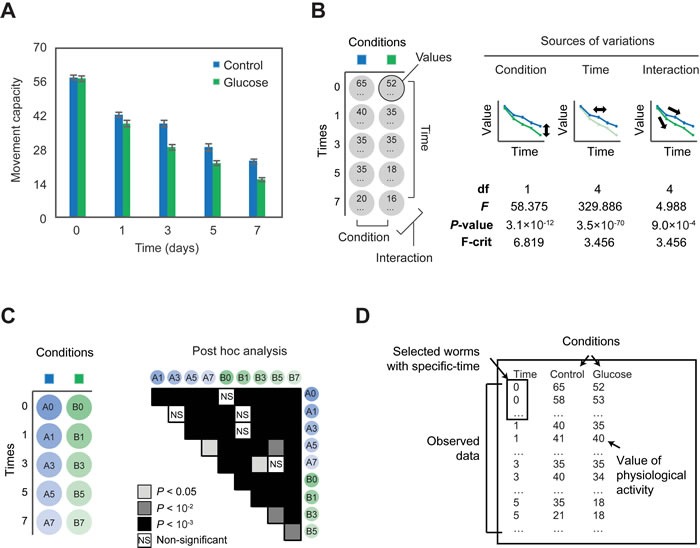
Analysis of differences in physiological values factored by time and conditions using OASIS 2 A Time-dependent changes in motilities of control and glucose-fed populations of wild-type *C. elegans*. **B.** Shown are conceptual figures of two-way ANOVA for testing differences in physiological-activity values among different times or conditions. Degrees of freedom (df) and the results of *F*-tests (*F*-value, *P*-value, and *F*-critical value) are displayed according to the sources of variation. **C.** Shown are the results of a post hoc analysis calculated by OASIS 2. Tukey's honest significant difference is used to calculate the *P*-value. **D.** Input for the analysis of physiological values.

OASIS 2 also provides statistical tools and graphical outputs for the comparison of values in multiple groups factored by condition and time, which are useful for analyzing differences in physiological activities depending on conditions and times. To demonstrate those features of OASIS 2, we re-analyzed data regarding the movement capacity of *C. elegans* worms of different ages, which we recently published [9]. Wild-type animals displayed declines in movement capacity during aging, which was accelerated by glucose-rich diets (Figure 2A). We implemented a two-way ANOVA that compares values in multiple groups factored by condition and time (Materials and Methods; Figure 2B). By using the ANOVA, we were able to show a significant difference in movement capacity between the control and glucose-treated animals (*P* = 3.1×10^−12^, Source of variation: Conditions; Figure 2B). We also showed a significant difference in movement capacity among worms of different ages (*P* = 3.5×10^−70^, Source of variation: Time). Furthermore, we showed that the effect of the glucose treatment depends on the age of the worm (*P* = 9.0×10^−4^, Source of variation: Interaction). For an additional exploration of the pair-wise differences among the means of multiple groups, we also performed a post hoc analysis implemented in OASIS 2 (Figure 2C).

OASIS 2 has an interface that generates graphical outputs for many kinds of changes in values (e.g., physiological activities) in multiple groups factored by conditions and times. The input data have a structured format that includes at least three columns: time and two conditions including a treatment and a control (Figure 2D). The columns should be divided with tabs. The users need to record the physiological activities of specific numbers of individuals from selected populations, which are in specific conditions and at specific time points (e.g., age).

**Figure 3:**
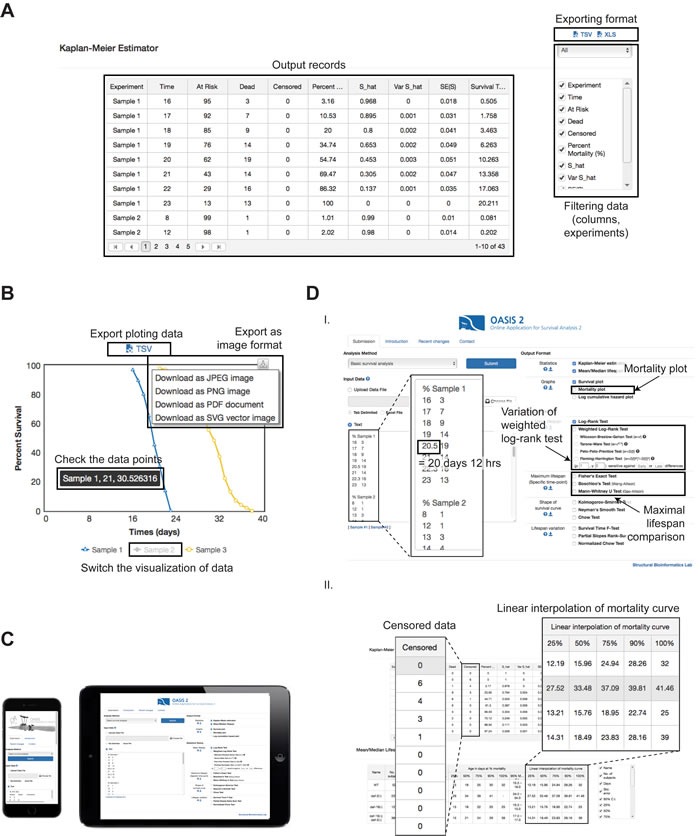
Features of the enhanced user-friendly interface of OASIS 2 **A.** Exporting options for data analysis in OASIS 2. Data filters are applied to the contents of both the output records and the exported data. **B.** Interactive charting of survival curves. Modified charts can be exported to various formats. Detailed data information can be highlighted as a popup when a mouse pointer hovers. The display of sample curves can be switched by clicking chart legends. **C.** OASIS 2 displayed in a smartphone (left) and a tablet-PC (right). **D.** User-friendly updates. Panel I: data submission page. The checkbox for drawing mortality plots is provided in the “Graphs” section. Panel II: output page. Counts of censored data are displayed in the results of a Kaplan-Meier estimation. Linear interpolation is added to the columns of the “Mean/Median Lifespan” section.

OASIS 2 gives a more user-friendly interface than the previous version. It enables the users to export the results of statistical analyses in various formats, in contrast to the previous version, which displayed the results only in HTML documents (Figure 3A). Moreover, users of OASIS 2 can customize the export options by choosing the combinations of columns in various formats. An interactive charting ability enables the users to compare survival curves more conveniently (Figure 3B). The interactive charts can be exported as modifiable vector images, such as Scalable Vector Graphics (SVG) and Portable Document Format (PDF) files, enabling users to easily obtain publication-quality figures. In addition, we updated the OASIS 2 webpage to follow the common web standards. Hence, users can operate OASIS 2 with tablets or smartphones as well as with personal computers (Figure 3C). OASIS 2 also works smoothly with various internet browsers including Microsoft Internet Explorer 11 and Edge, Google Chrome, and Apple Safari on Windows and Mac OS X.

We also updated OASIS 2 to adapt to the needs of experimental biologists (Figure 3D). Users can analyze survival data not only on a daily basis but also on an hourly one, because OASIS 2 now supports numbers with decimal points for time inputs. For example, 20.5 days indicates 20 days and 12 hours. Other features updated for user convenience include (1) mortality-rate plotting, (2) linear interpolation of the mortality curve, and (3) the counting of censored data. For researchers who need to analyze security-sensitive data, such as data related to the Health Insurance Portability and Accountability Act, OASIS 2 has two features to ensure data security. First, analyzed data can be accessed only by those with the IP addresses of the data uploaders. Second, the uploaded data are automatically discarded after 10 days.

OASIS 2 also provides several weighted log-rank tests that can be used for various statistical tests. OASIS 2 now supports the Wilcoxon-Breslow-Gehan test, Tarone-Ware's test, and the Peto-Peto-Prentice test as well as Fleming-Harrington's test (Figure 3D). We also validated those statistical tests and confirmed that the results of OASIS 2 are same as the results obtained from the statistical package STATA (http://sbi.postech.ac.kr/oasis2/benchmark#wlogrank).

## DISCUSSION

Recently, web-based tools such as OASIS and SurvCurv have emerged as convenient analysis tools for survival data. In this paper, we report on the new features of OASIS 2, which provides advanced statistical tools and an enhanced user interface. OASIS 2 provides features such as the statistical comparison of maximal lifespan. It has recently emerged that physiological changes with age have been used to measure healthspan in model animals as well as in humans [10, 11]. Thus, the analysis pipeline for values in multiple groups factored by condition and time can provide useful tools for the analysis of healthspan, which relies on the analysis of changes in physiological activities during aging. We believe that OASIS 2 will help experimental biologists to easily conduct various statistical analyses associated with the complex phenomena of aging.

## MATERIALS AND METHODS

### *C. elegans* data set

Lifespan assays were performed as described previously [12]. Briefly, wild-type (N2) and *fat-6*-overexpressing worms (IJ508 *yhEx112 [ges-1p::fat-6::GFP, odr-1::RFP]*) were maintained on *Escherichia coli* (OP50)-seeded nematode growth medium (NGM) agar plates at 20°C. Synchronized wild-type and *fat-6*-overexpressing animals were transferred onto OP50-seeded NGM agar plates containing 10 μM 5-fluoro-2′-deoxyuridine (FUdR, Sigma, St Louis, MO, USA) at day 1 adult stage to prevent progeny from hatching. Worms that did not respond to gentle touching by a platinum wire were counted as dead. Animals that crawled off the plates, ruptured, or burrowed were censored but included in the statistical analysis. The movement-capacity data were adopted from our previously published paper [9].

### Statistical analysis of maximal lifespan comparison

Modifications of Boschloo's Test and the Mann-Whitney *U* Test were adopted to implement the comparisons of maximal lifespans in OASIS 2. The modifications of each test were designed by Gao-Allison and Wang-Allison, respectively [6, 13]. The null hypothesis of Boschloo's test (*H*_0, A_) is that the fraction of outliers that live beyond specific time points is similar between populations A and B. The equation of *H*_0, A_ is:

$$H_{0,A}:P(L(x)>\tau |x\in A)=P(L(x)>\tau |x\in B),$$

where *x* is an observation from the population, *L*(*x)* is the survival time of *x*, and t denotes some threshold chosen by the investigator, which could represent the criteria for a specific time point. We used the 90th percentile threshold as t. Boschloo's test is applied to compare the fractions of outliers. The null hypothesis of the Mann-Whitney *U* test (*H*_0, AB_) is the compound of *H*_0, A_ and another null hypothesis (*H*_0, B_). *H*_0, B_ is that the outliers have a similar average survival time between two different populations. The equation of the compound null hypothesis (*H*_0, AB_) is:

$$\begin{array}{c} H_{0,AB}:\mu (Z(x)|x\in A)=\mu (Z(x)|x\in B),\\ Z(x)\equiv I(L(x)>\tau )\cdot L(x), \end{array}$$

where *I*(*o*) is an indicator function taking on a value of 1 if *o* is true and a value of 0 otherwise. The Mann-Whitney *U* test is applied to compare the average (μ) of *Z* variables between two populations.

### Statistical comparison of physiological activity

ANOVA is used to analyze differences in physiological activities depending on condition and time. Among different conditions, the mean values should change if the values are affected by the conditions over time. Therefore, the null hypothesis of the test (*H*_0_) is that the means of all samples are equal regardless of the variation in conditions. The equation of *H*_0_ is:

$$H_{0}:\mu_{1}=\mu_{2}=\mu_{3}=\cdots =\mu_{k^{\prime }},$$

where μ_1_, …, μ*_k_* are the means of each condition. The *H*_0_ is rejected when at least one pair of means is not equal. To test the *H*_0_, the *F*-test is used. The *F*-value is calculated from the following formula:

$$F=\frac{MS_{condition}}{MS_{error}}=\frac{\frac{SS_{condition}}{\left(k-1\right)}}{\frac{SS_{error}}{k(n-1)}},$$

where *MS* is the mean square, *SS* is the sum of squares, *k* is the number of conditions, and *n* is the number of samples within each condition. Finally, the *P*-value is estimated from the *F*-distributions.
